# Allocating external financing for health: a discrete choice experiment of stakeholder preferences

**DOI:** 10.1093/heapol/czx017

**Published:** 2018-02-05

**Authors:** Karen A Grépin, Crossley B Pinkstaff, Arne Risa Hole, Klara Henderson, Ole Frithjof Norheim, John-Arne Røttingen, Trygve Ottersen

**Affiliations:** 1 Department of Health Sciences, Wilfrid Laurier University, 75 University Ave, W. Waterloo, ON N2L3C5, Canada,; 2 International Food Policy Research Institute, 2033 K Street, N.W. Washington, D.C. 20006, U.S.A,; 3 University of Sheffield, Sheffield, UK,; 4 Independent Consultant, Warringah Street, North Balgowlah, NSW 2093, Australia,; 5 University of Bergen, Bergen, Norway,; 6 Infectious Disease Control and Environmental Health, Norwegian Institute of Public Health, Lovisenberggata 8, 0456 Oslo, Norway, Department of Global Health and Population, Harvard T.H. Chan School of Public Health, Harvard University, 655 Huntington Ave, Boston, MA 02115, USA, Department of Health Management and Health Economics, University of Oslo, Forskningsveien 3a/2b, 0373, Oslo, Norway and; 7 Department of International Public Health, Norwegian Institute of Public Health, Norway, Marcus Thranes gate 2, 0473 Oslo, Norway, Oslo Group on Global Health Policy, Department of Community Medicine and Global Health and Centre for Global Health, University of Oslo, Kirkeveien 166, 0450 Oslo, Norway, Department of Global Public Health and Primary Care, University of Bergen, Kalfarveien 31, 5018 Bergen, Norway

**Keywords:** Development assistance for health, health policy, discrete choice, priority setting, health politics

## Abstract

Most donors of external financing for health use allocation policies to determine which countries are eligible to receive financial support and how much support each should receive. Currently, most of these policies place a great deal of weight on income per capita as a determinant of aid allocation but there is increasing interest in putting more weight on other country characteristics in the design of such policies. It is unclear, however, how much weight should be placed on other country characteristics. Using an online discrete choice experiment designed to elicit preferences over country characteristics to guide decisions about the allocation of external financing for health, we find that stakeholders assign a great deal of importance to health inequalities and the burden of disease but put very little weight on income per capita. We also find considerable variation in preferences across stakeholders, with people from low- and middle-income countries putting more weight on the burden of disease and people from high-income countries putting more weight on health inequalities. These findings suggest that stakeholders put more weight on burden of disease and health inequalities than on income per capita in evaluating which countries should received external financing for health and that that people living in aid recipient may have different preferences than people living in donor countries. Donors may wish to take these differences in preferences in mind if they are reconsidering their aid allocation policies.

## Introduction

Most low- and middle-income countries (LMICs) receive some external financing for health, including development assistance for health (DAH) and other forms of assistance, from donor agencies and other international actors to support the delivery of health services. Since 2000, there has been a rapid increase in the annual level of DAH provided by donors (Ravishankar *et al.* 2009). Increased external financing has been driven by increased aid from bilateral donors, the establishment of new multilateral agencies (e.g. Gavi), and from the rise of global health philanthropies (e.g. the Bill and Melinda Gates Foundation) (Murray *et al.* 2011). However, since 2010, commitments of DAH from major donors have plateaued (Dieleman *et al.* 2015), leading to calls for improving the prioritization of external financing for health to generate more “value for the money”. For example, donors have increasingly discussed the need to increase the efficiency of existing programs (Grépin 2012a). Others have called for donors to allocate external financing in a way that more closely aligns with the global burden of disease (Dieleman *et al.* 2014). And a Center for Global Development working group has called on donors to prioritize programs on the basis of cost-effectiveness, which could save more lives and promote equity (Glassman and Chalkidou 2012).

Most donors of external financing for health use some sort of allocation policy to determine which countries are eligible to receive financing as well as how much financing they should be allocated (Ottersen *et al.* 2017). Here, we define an allocation policy as an explicit or implicit rule used to determine both whether a country is eligible to receive external financing as well as the amount of financing that a country receives. Most global health donors currently use GNI per capita as the basis of their allocation policies but some also include additional criteria, such as those related to health needs or aid effectiveness (Ottersen *et al.* 2017). Several of these allocation policies are partly expressed in terms of explicit formulae, in which the amount of aid allocated is a function of one or more country indicators, weighted to reflect donor preferences.

The evolving landscape of external financing for health has led many global health donors to rethink their allocation policies, which is in essence a re-prioritization exercise. There is a general agreement that the primary intent of external financing for health is to improve health in countries where government resources are insufficient to fully fund priority health programs. Income per capita is used as a primary determinant of most allocation policies due mainly to the fact that it is available for all countries and because it is seen as a good proxy for overall level of health and development across countries (Fantom and Serajuddin 2016). But while it is a useful general indicator, it may be inadequate for guiding the allocation of external financing for health. One reason for this is GNI per capita is not perfectly inversely correlated with population health needs. Another reason is that GNI per capita only reflects average income and not its distribution, which means that inequalities are not directly captured.

As part of an initiative called the Equitable Access Initiative (EAI, please see http://www.theglobalfund.org/en/equitableaccessinitiative/ for more information on this initiative), the authors of this article were involved in a broader research effort that included four technical teams that were all tasked with developing a new approach to classify countries with regards to decisions for the allocation external financing for health Ottersen *et al.* 2017b; Ottersen *et al.* 2016. The co-convening partners of the EAI included the World Health Organization, Gavi, The Vaccine Alliance, UNAIDS, UNICEF, UNDP, UNFPA, UNITAID and the Global Fund.

All of the frameworks and methodologies proposed by the technical teams involved identifying additional criteria beyond income per capita and then combining these criteria together in some way to generate overall country rankings to guide aid allocation decisions. A key open question that arose during the EAI process was that in order to combine different criteria into a single equation or formulae, whether implicitly or explicitly, donors need to determine how much emphasis, or weight, to put on any given criteria in their overall assessment of countries. It also became apparent through this process that different stakeholders had different preferences for how much weight should be placed on each of these country-level criteria in decisions about external financing for health and it therefore was unclear how these criteria should be combined.

One strand of the academic literature has investigated the determinants of existing aid allocation patterns across donors. In the general aid allocation literature, while income per capita is almost always negatively associated with the amount of aid a country receives, studies have found that other factors also influence aid allocation, including political factors (Alesina and Dollar 2000; Kuziemko and Werker 2006) and quality of governance in aid recipient countries (Dollar and Levin 2006). Within the health sector, a few studies have also investigated the factors that influence DAH allocation patterns. One study that looked at official DAH commitments from 2005-07, found that burden of disease measures only weakly predicted how much health aid a country received (Esser 2011). Another study found that after controlling for income per capita and burden of disease, measures of political rights and levels of corruption also predicted aid allocation patterns (Fielding 2011). A more recent study has found that global health donors do respond to the health needs of a country in that countries with higher levels of infant mortality, child mortality and HIV mortality receive more health aid, controlling for donor and recipient government level factors, than other countries (Lee and Lim 2014). While this literature can be useful in terms of informing the discussion of aid allocation, the EAI was motivated by a desire to answer the question about how global health donors should prioritize aid across countries and not necessarily how aid is currently allocated.

One methodology that can be used to elicit preferences for deriving weights across country criteria is a discrete choice experiment (DCE). This is a quantitative technique to empirically elicit respondents’ stated preferences over choice alternatives with different characteristics (Ryan and Gerard 2008). DCEs have gained popularity in the health economics literature over the past 20 years. They have also increasingly been used to elicit preferences to inform health policy and priority-setting questions in LMICs (Mangham *et al.* 2009). DCEs provide respondents with a series of hypothetical choice sets and ask respondents to choose their preferred alternative from within each choice set. By making a choice, it is believed that respondents are revealing their true preferences over the characteristics of the alternatives. DCEs have roots in random utility theory, which assumes that the respondent evaluates alternatives based on their utility for him or her and then selects the one that provides the most utility.

There have been relatively few studies of stated preferences regarding the allocation of aid across countries. Using conjoint analysis, Bachke *et al.* (2013) polled a sample of Norwegian university students and found that different types of respondents have different preferences for how aid should be allocated, for example with different preferences among male and female respondents. In a similar study, Cunningham *et al.* (2016) found that university students in New Zealand rank aid effectiveness highly, almost on par with aid programs that prioritize country need. Hansen *et al.* (2014) also find that university students in New Zealand rank concerns such as health needs and infrastructure more highly than income per capita in deciding which countries to donate money to. To our knowledge, no studies on the allocation of aid have elicited the preferences of people from both LMICs and high-income countries, and no studies have elicited the preferences of real-world stakeholders, rather than just university students. Moreover, no study, to our knowledge, has specifically elicited preferences for the allocation of external financing for health across countries.

The purpose of this article is to report on a survey that elicited preferences of different stakeholders for criteria guiding the allocation of external financing for health across countries using an online DCE. We first provide a brief summary of the methods, before analysing the results of the DCE, and then discuss the implications of these findings for the development of new frameworks for ranking countries for the allocation of external financing for health.

## Methods

To elicit preferences, we developed a DCE that we administered as an online survey using Qualtrics (www.qualtrics.com). A key element of the design of any DCE is the choice of attributes, or country characteristics, to be included into the experiment. To identify these attributes, we first conducted a background review on the motivations for aid allocation in the academic literature. In addition, we also conducted 20 key informant interviews with stakeholders involved with the EAI, including representatives of the EAI co-convening partners, representatives of both donor and receiving countries, and civil society organizations, about what they perceived as the key criteria that should be used in ranking countries for health aid allocation purposes (Ottersen *et al.* 2016). Based on this background research and our interviews, we developed a list of potential country criteria. In order for the survey to be easy for the respondents to completed, we then selected four criteria that were commonly cited in our background research, that we believed would be easily understood by the survey respondents, and which were as mutually exclusive from one another as possible. The four country criteria we selected were: income per capita, overall burden of disease, strength of the health system, and level of health inequality. Prior to launching the DCE, we also presented these four criteria during the EAI consultation process to get feedback prior to launching the survey.

**Figure 1.**
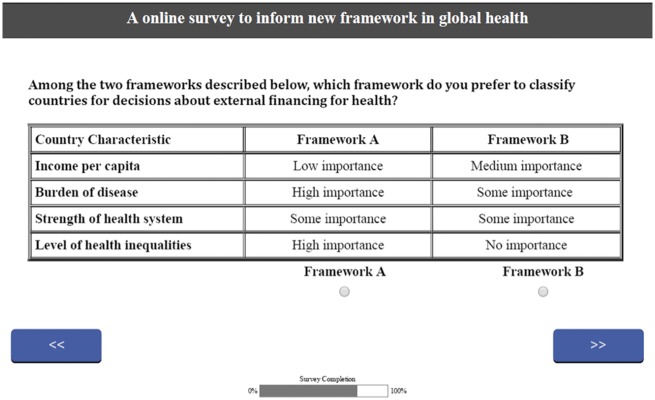
Example of one of the choice sets presented to stakeholders

For each country characteristic, we then defined the levels of importance for each. For income per capita, since level of income is the primary determinant of which countries received aid, we decided that it was unlikely that any respondent would not put any emphasis on income per capita in the allocation of aid, so for it the choices were low, medium and high levels of importance. For the other criteria, we defined three levels of importance for each criteria: no, some or high level of importance. Based on our choice of country characteristics and levels of importance, we ended up with an experimental design that included 81 possible frameworks (3 × 3 × 3 × 3). This leads to a very large number of potential pairs of frameworks to present to each respondent using paired choice sets, which was deemed to be impractical (Lancsar and Louviere 2008). To simplify the choices, we developed an orthogonal main-effects plan with high levels of balance and minimal overlap to reduce the number of questions each respondent needed to answer down to 9 (Huber and Zwerina 1996; Street *et al.* 2005). An example choice from the survey is shown in Figure 1.

Respondents were also asked to answer background questions about their age, gender, country of birth, highest level of education completed and type of organization in which they worked. Some variables were recoded as dummy binary variables (male vs female, born in a high vs LMIC) to simplify the analysis. All respondents were asked to provide informed consent and this research component received ethical approval from our institute. Please see the Supplementary Appendix (Supplementary data are available at *Health Policy and Planning* online) to this paper for more details and specifics of the survey itself.

The data from the DCE were used to estimate a mixed logit model, which is an appropriate method for modelling data on discrete choices between two or more alternatives (Ryan and Gerard 2008). All of the analysis was carried out in Stata using the mixlogit command (Hole 2007).

The EAI convening partners assisted us with generating a sample of respondents for the survey. From the Global Fund, we received a list of e-mail addresses of people who had recently expressed interest in participating in a regional partnership forum or an online eForum at the Global Fund. The majority of people on this mailing list were either members of civil society or were practitioners involved in health aid-related activities in LMICs. For the other convening partners, EAI focal points were e-mailed and asked to forward the survey to members of their organization with a request to complete the survey. We sent ∼1500 e-mail invitations, but are unable to know exactly how many people received an invitation to complete the survey.

## Results

A total of 285 people consented and completed the survey. Slightly less than half of the sample (45.3%) was female. The sample was highly educated, with only 19.3% reporting an undergraduate degree or less. The sample had broad geographic coverage with responses coming from almost 90 countries. Respondents were most likely to have been born in a high-income country (35.8%), followed by a lower middle-income country (34.0%), a low-income country (15.8%) and an upper middle-income country (14.8%). Civil society organizations were well represented in the sample—accounting for 44.6% of the respondents. International organizations and academic/consultant institutions were also well represented (28.8 and 11.2%, respectively). An equal share of respondents worked for aid donor and recipient governments, with 4.6% for each. See Table 1 for a full description of the sample.

**Table 1.** Summary statistics of sample

Completed responses	*N*	%		
	285	100		
Gender				
Female	129	45.3		
Education				
Undergraduate or less	55	19.3		
Graduate degree	149	52.3		
Medical degree	33	11.6		
PhD	36	12.6		
Other or unknown	12	4.2		
Organizational affiliation				
Civil society organization	127	44.6		
International organization	82	28.8		
Academic/commentator/consultant	32	11.2		
Government receiving external assistance	13	4.6		
Government providing external assistance	13	4.6		
Industry	4	1.4		
Other	14	4.9		
**Location of respondents**	**By country of birth**		**By country of residence**	
Total number of countries	89		88	
High Income	102	35.8%	103	36.1%
Upper Middle Income	41	14.4%	40	14.0%
Lower Middle Income	97	34.0%	97	34.0%
Low Income	45	15.8%	45	15.8%

Our primary findings are summarized in Table 2. The relative magnitudes of the mean coefficients indicate the relative importance attributed to each of the levels of each country characteristics in the full sample of responses, on average. Overall, the strongest predictor of framework choice was whether the framework assigned high importance to the level of burden of disease or health inequalities. In other words, a framework that placed high importance for burden of disease or health inequalities had a greater chance of being chosen than a framework without this property (holding all other characteristics constant). Beyond these criteria, positive but less influential determinants of framework choice included some level of importance assigned to either burden of disease or health inequalities, and high importance assigned to strength of the health system. Respondents placed much less weight on how much importance the frameworks assigned to income per capita. The estimated standard deviations in the mixed logit models indicate that there is significant preference heterogeneity among the respondents for the high importance levels of each country characteristic. This indicates that individual respondents’ preferences can deviate markedly from the general trend described above, and suggests that stakeholders differ in their preferences for any model that puts a high level of weight on any one of these country criteria. In addition, the alternative-specific constant for Framework A is negative and significant, which shows a preference for Framework B over Framework A, holding the country characteristics constant. This is unexpected, as there is no reason to prefer one framework over another on any other basis than the country characteristics. Dropping the alternative-specific constant from the model, however, did not have a qualitative impact on the results.

**Table 2.** Importance of attributes in framework choice, full sample

Attribute	Mean		Standard deviation	
	Coefficient	*P*-value	Coefficient	*P*-value
Country income (omitted: low importance)				
Medium importance	0.27	0.03	0.05	0.87
High importance	0.21	0.11	0.81	0.00
Burden of disease (omitted: no importance)				
Some importance	1.26	0.00	0.01	0.98
High importance	1.86	0.00	1.56	0.00
Strength of health system (omitted: no importance)				
Some importance	0.67	0.00	0.09	0.76
High importance	1.12	0.00	0.59	0.00
Level of health inequality (omitted: no importance)				
Some importance	1.08	0.00	0.22	0.48
High importance	1.80	0.00	1.04	0.00
Alternative-specific constant				
Framework A	−0.14	0.02	0.28	0.07
Number of observations	5130			
Number of responses	2565			
Number of respondents	285			
Number of responses per respondent	9			
Log likelihood	−1399.88			
Pseudo *R*^2^	0.21			

Notes: All random coefficients are specified to be normally distributed, and the coefficients reported in the “Mean” and “Standard deviation” columns report the estimated moments of the distribution. 500 Halton draws were used to approximate the log-likelihood function in the simulated likelihood procedure.

In Table 3, we also show how framework choices varied by whether the respondent was born in a high-income country or an LMIC (according to the World Bank classification for 2014). These groupings of countries were selected due to the fact that the former are typically aid donor countries while the latter are typically aid recipient countries. Respondents born in what is now an LMIC were most influenced by whether the frameworks assign high importance to burden of disease, followed by high importance to level of health inequalities, and some importance to burden of disease. In contrast, respondents who were born in a high-income country ranked high importance of health inequalities as the most important country characteristic, followed by high importance of the burden of disease and some importance of health inequalities. Neither group put a great deal of weight on the importance of income per capita. As in the pooled sample there is evidence of significant preference heterogeneity among the respondents in each group. The alternative-specific constant is negative and significant for the LMIC group, which is unexpected for the reasons discussed above, while it is small and insignificant for the high-income group in line with our expectations.

**Table 3.** Importance of attributes for framework choice, sample split by income level of country of birth

Attribute	High Income Country				Low and Middle Income Country			
	Mean		Standard deviation		Mean		Standard deviation	
	Coefficient	*P*-value	Coefficient	*P*-value	Coefficient	*P*-value	Coefficient	*P*-value
Country income (omitted: low importance)								
Medium importance	0.24	0.28	0.34	0.49	0.29	0.06	0.34	0.13
High importance	0.03	0.91	1.44	0.00	0.32	0.06	0.60	0.00
Burden of disease (omitted: no importance)								
Some importance	1.34	0.00	0.07	0.84	1.36	0.00	0.06	0.84
High importance	1.98	0.00	1.94	0.00	2.04	0.00	1.59	0.00
Strength of health system (omitted: no importance)								
Some importance	1.47	0.00	0.09	0.88	0.35	0.11	0.05	0.90
High importance	1.42	0.00	0.53	0.18	1.10	0.00	0.81	0.00
Level of health inequality (omitted: no importance)								
Some importance	1.61	0.00	0.17	0.85	0.94	0.00	0.37	0.14
High importance	2.47	0.00	1.13	0.00	1.70	0.00	1.19	0.00
Alternative-specific constant								
Framework A	0.01	0.91	0.22	0.56	−0.23	0.00	0.39	0.02
Number of observations	1836				3294			
Number of responses	918				1647			
Number of respondents	102				183			
Number of responses per respondent	9				9			
Log likelihood	−478.20				−901.57			
Pseudo *R*^2^	0.25				0.21			

Notes: All random coefficients are specified to be normally distributed, and the coefficients reported in the “Mean” and “Standard deviation” columns report the estimated moments of the distribution. 500 Halton draws were used to approximate the log-likelihood function in the simulated likelihood procedure.

**Figure 2.**
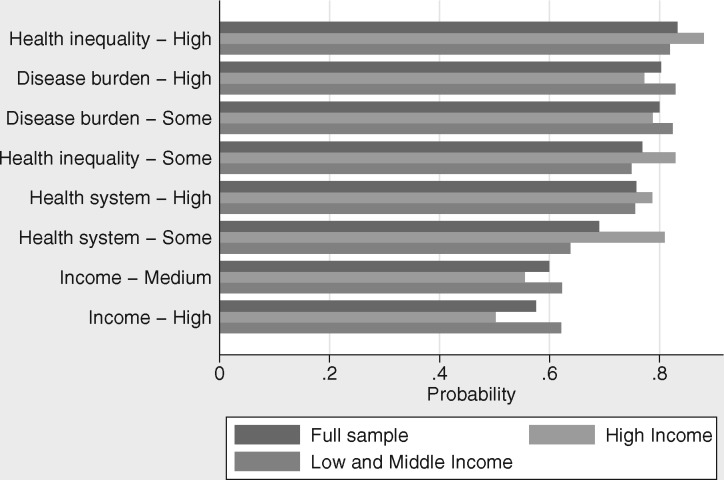
Predicted probabilities for full and split samples

Figure 2 shows the predicted probabilities generated from the mixed logit models presented in Tables 2 and 3. There are two competing frameworks, for which the attribute levels have been set as “No/low importance” for all attributes except one for Framework B, which has the level shown in the figure. For example in the first row, “health inequality—high”, shows the probability of choosing a framework that has “No/low importance” for income, health system and disease burden and high importance for health inequality (Framework B), over another framework that has “No/low importance” for all of the attributes (Framework A). The figure presents the predicted probabilities for the full sample, as well as for people from LMICs and high-income countries separately. The predicted probabilities reveal the same overall preference pattern as the estimated coefficients, but with the added benefit of having a straightforward quantitative interpretation.

## Discussion

This DCE elicited preferences for frameworks for the allocation of external financing for health that differ in the importance they assigned to each of four country characteristics. Overall, the findings suggest that our surveyed stakeholders rank the country characteristics in the following order of decreasing importance: level of health inequality, burden of disease, strength of health system and income per capita. The findings also suggest that different stakeholders differ in their preferences, in particular, respondents from LMICs attributed lower importance to inequality and strength of the health system than respondents from high-income countries. However, both groups of respondents ranked income per capita as the least important characteristic. In addition, we also observed high levels of unobserved heterogeneity in preferences for all frameworks that put high importance to any of the country characteristics tested. These findings can inform policies and decisions on eligibility and allocation in multiple ways.

First, discrepancies between current policies and our observed preferences of stakeholders give reason to reconsider current external financing for health allocation policies. We found that respondents are most concerned with health inequalities and disease burden and least concerned with income per capita. This is at odds with current policies for eligibility and allocation, which tends to emphasize income per capita. Gavi, the Global Fund, the World Bank, UNICEF and UNDP all assign GNI per capita a central role in classifying countries and allocating funds (Ottersen *et al.* 2017). Of the large multilateral donors in global health, the Global Fund is the one that adjusts GNI per capita most explicitly for disease burden in classifying countries and determining eligibility, however it was not until 2013 that it gave disease burden this significant role (Kapilashrami and Hanefeld 2014). To our knowledge no donor explicitly uses measures of health inequality in their allocation policies. The findings from the survey thus give most donors reason to reconsider their policies for the eligibility and allocation of external financing for health. In particular, the survey provides reason to examine if GNI per capita gets too much weight and health inequalities and disease burden too little. However, variation in preferences for all of the models with high importance suggests that any model that dramatically shifts weight to a single criteria is also unlikely to fit the preferences of all stakeholders.

Second, variation across groups of respondents gives reason to reconsider whose values and preferences should guide external financing for health. In particular, we found significant differences in preferences across respondents from LMICs and high-income countries. Since donors tend to come from wealthier countries and those on the receiving end of external financing tend to be from poorer countries, this finding provides concrete input to discussions about aid alignment (Sridhar 2010). Donors have been criticized for giving too much priority for donor priority programs at the expense of health systems or national health priorities (Grépin 2012b). On the basis of the findings from this study, donors may wish to systematically consider the preferences of people living in LMICs in the redesign of their allocation policies, for example through the use of public opinion polls or an additional survey building off this study.

Third, by being one of the first of its kind, this study provides a basis for further inquiry into preferences for the allocation of external financing for health. Future studies should attempt to address some of the limitations of this study. First, like all stated-preference studies, our study results are sensitive to the framing of the questions and choices we used, including the fact that this was an online survey. Second, also like all stated-preference studies, our findings are potentially sensitive to which country characteristics were included in the survey. Being the first study of its nature, there were no previous studies to use to help guide the choices of country characteristics. While we used a review of the literature as well as interviews and consultations with stakeholders to identify these characteristics, the selection of characteristics was still somewhat arbitrary. Future research should attempt to first pilot additional criteria and to test the importance of other characteristics as well.

Fourth, for simplicity, we used relatively crude attribute levels (low/no, some/middle, high), which may be interpreted differently by different stakeholders and may have influenced our results. The significant standard deviation on all high importance coefficients may be due to the broad attribute levels. Future studies could use more fine-grained levels, but this must be balanced against other uses of respondents’ time. On a related note, although we consulted with many stakeholders prior to conducting the surveys, we did not get the chance to conduct a proper pilot to see how the choice of attributes and levels influenced the preferences of stakeholders. It is possible, for example, that the difference in the way in which we described the importance of income per capita may have influenced the low priority it obtained in all of the methods. Fifth, the presence of a significant alternative-specific constant among respondents from LMICs, but not in HICs, may be due to differences in the way in which the survey was understood or completion rates among respondents from these different areas. Future research should also attempt to better understand these potential effects and should consider the inclusion of an opt-out or status-quo alternative, instead of using a forced-choice design.

Sixth, we were not able to directly control what types of people answered the survey and our results may have differed if we had been able to obtain responses from a more representative sample. That said, it is unclear exactly what the most appropriate population is in this context. We did get very high levels of geographic coverage in our sample, making our sample more heterogeneous than employed in previous studies. However, our sample was very highly educated and it is likely that less educated people would have different preferences. Certain categories of stakeholders were also unlikely to be reached through the method of recruitment used. This includes the most vulnerable groups and populations and those with the greatest needs. In future research, a more tailored approach to these stakeholders should be used. If alignment with stakeholder preference is a goal, more research could help to elucidate on how to improve policy and practice.

Finally, some of the categories we used to describe the respondents were not completely mutually exclusive and a more detailed list of organizations could have been useful.

## Conclusion

There is growing awareness of the need to carefully prioritize external financing for health across countries. In particular, there is an increasing understanding of the need to go beyond GNI per capita alone when classifying countries and allocating health aid. The findings of this study reinforce the view that most stakeholders want to move beyond GNI per capita as the primary determinant of health aid eligibility and allocation. The findings also suggest that if donors want to supplement GNI per capita, health inequalities and disease burden are key candidates for consideration. The findings of this study can thus inform ongoing policy discussions and the quest for sufficiently nuanced frameworks for classifying countries and allocating assistance.

## Ethical approval

Exemption from full IRB approval was obtained from the Human Subjects Office at New York University prior to data collection. Human subjects were provided informed consent.

## Funding

This work was supported by the Wellcome Trust [099114/Z/12/Z].

*Conflict of interest statement*. None declared. 

## Supplementary Material

Supplementary Appendix
