# Supplementary material for: Allocating external financing for health: a discrete choice experiment of stakeholder preferences
Source: Health Policy Plan. 2018 Feb 5;33(Suppl 1):i24–30. doi: 10.1093/heapol/czx017 (PMC5886273; doi:10.1093/heapol/czx017)
Supplement: Supplementary Appendix [file czx017_dce_appendix_2resubmit.docx]

**Appendix to “Allocating external financing for health:**

**a discrete choice experiment of stakeholder preferences”**

The following appendix provides more detailed information on some of the specifics of the design of the discrete choice experiment described in the accompanying manuscript as well as additional analysis that were not presented in the main manuscript.

**Appendix Section 1: Additional Details of the Survey**

The discrete choice experiment outlined in this manuscript was administered via an online survey in the fall of 2015. Respondents were contacted via email and asked to complete the online survey. The following was used as the text to introduce the survey to the respondents. Author specific information has been removed from the text below but was included in the original survey.

***Introduction to the Survey***

The [Equitable Access Initiative](http://www.theglobalfund.org/en/equitableaccessinitiative/) (EAI) is building a new policy framework to better understand the health needs and constraints of countries as they move along the development continuum.

As part of the process, the EAI has engaged the XXXX to conduct research to inform a new framework in global health, which could be used for classifying countries in terms of characteristics relevant to guiding decisions on external financing for health. The following survey has been designed to better understand which country characteristics various stakeholders believe are important and how they could be included into such a framework. The XXXX team is independent, in that the Convening Partners are neither responsible for the content of the survey, nor responsible for taking the responses into account.

Should you have any questions regarding this survey, or if you have any problems completing the survey, please do not hesitate to contact XXX, XXXX (XXX@XXX.edu).

***Informed Consent***

You have been invited to take part in a research study about preferences for global health frameworks to guide decisions for external financing for health. This study will be conducted by XXXX, in collaboration with researchers at the XXXX.

If you agree to be in this study, you will be asked to do the following:

1. Provide some demographic information

2. Complete a short online survey

Participation in this study will take 5-10 minutes.

There are no known risks associated with your participation in this research beyond those of everyday life. Although you will receive no direct benefits, this research may help the investigator understand preferences for global health frameworks to guide decisions for external financing for health.

Confidentiality of your research records will be strictly maintained by not collecting any personally identifiable information and all data will be securely stored.

Participation in this study is voluntary. You may refuse to participate or withdraw at anytime without penalty. Nonparticipation or not completing the survey will not affect you in anyway, however your responses will not be included in the findings of this study.

If there is anything about the study or your participation that is unclear or that you do not understand, if you have questions or wish to report a research-related problem, you may contact XXXX at XXXX or XXXX.

For questions about your rights as a research participant, you may contact the XXXX, at XXX@XXX.edu or by phone at XXX.

Please select one of the following options. If you choose not to participate, the survey will end immediately. [I agree/I do not agree]

***Survey Description Page***

The following survey is designed to elicit your preferences for a new framework that could be used for classifying countries in terms of characteristics relevant to guiding decisions on external financing for health.  In the survey, you will be presented with 9 pairs of frameworks.  In each pair, frameworks differ in terms of how much importance each places on particular country characteristics. More importance suggests that the framework puts more emphasis on the characteristic when classifying countries for guiding decisions about external financing for health.

The country characteristics chosen have been informed by a review of the literature and by discussions with various stakeholders. They are intended to capture the concerns that many of us in global health share: the level of economic development, the burden of disease, the strength of the health system, and the level of health inequality that exists within a country.

You may or may not believe that any or all of these characteristics should inform decisions regarding external financing for health.  We however ask that you select which framework you prefer within the pair – it need not be the framework you would prefer overall. However, at the end of the survey, we will also ask you about your overall preferences.

The survey is based on a discrete choice experiment so it may seem that some of the questions are repetitive. This is by design. We ask that you answer all of the questions based on what you personally believe rather than what you think follows from your professional affiliation.

You must complete each question before moving to the next screen, however, you may use the back arrow to scroll through your previous responses.

The survey should take 5–10 minutes to complete

**Screen Shot of Framework Choice Screens**

**
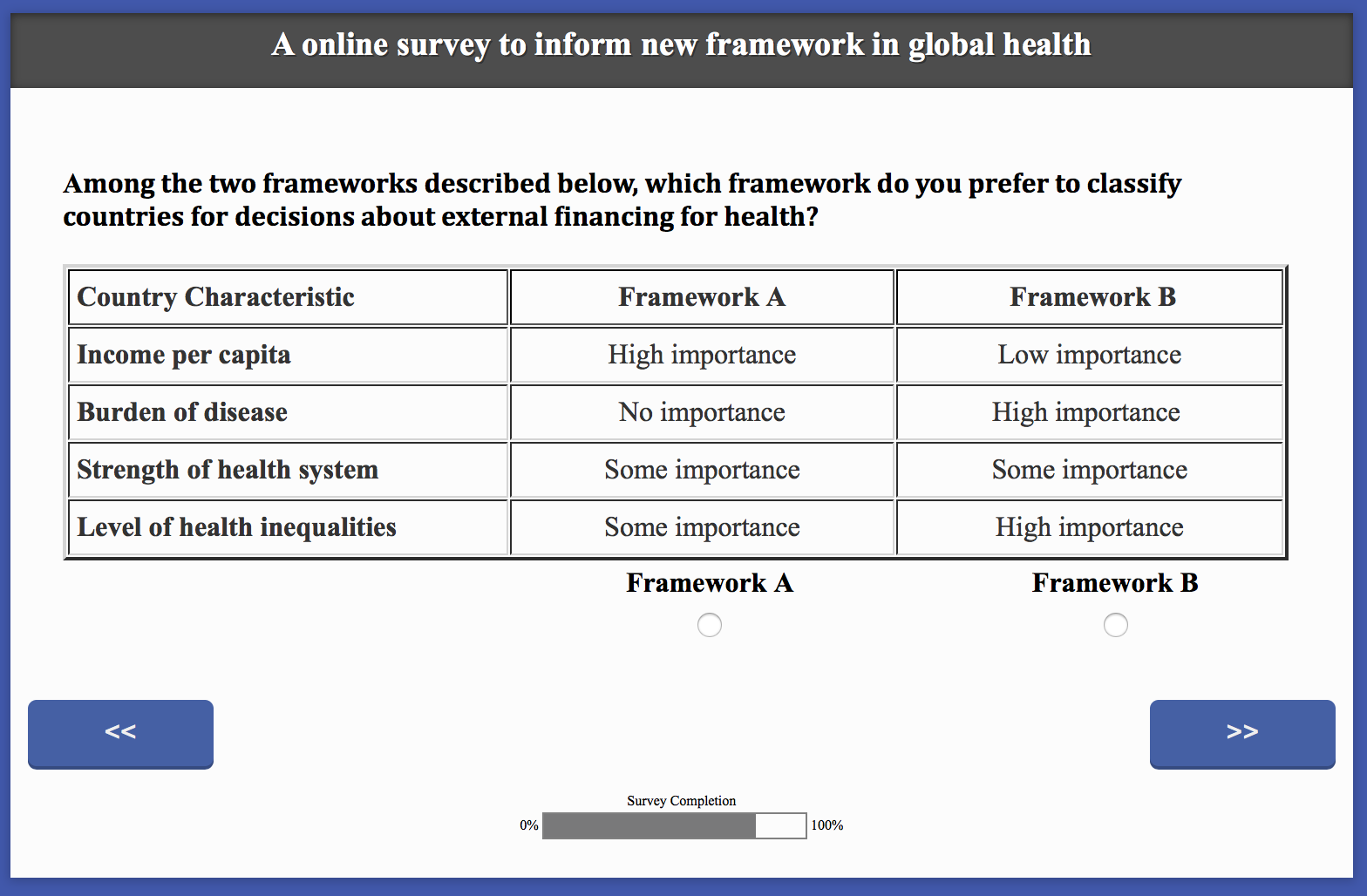
**

Figure 1: A sample screen shot of one of the choice sets

**Demographic Questions with possible choices:**

1. What is your age (continuous choice)?
2. What is your gender?
   1. Male
   2. Female
   3. Transgender
3. In which country do you live (pull down menu with list of countries)?
4. In which country were you born (pull down menu with list of countries)?
5. What is the highest level of education you have completed? While all of the categories were presented in the survey, categories a. and b. were collapsed in Table 1 due to low numbers of each. It did not affect our analysis.
   1. Less than undergraduate
   2. Undergraduate degree (College/University)
   3. Graduate degree
   4. Medical degree
   5. PhD
   6. Other (please specify)
6. Which best describes the organization for which you work or are a member of? While all of the following choices were presented in the survey, categories a. and b. were collapsed and categories g. and h. were collapsed in Table 1 due to low numbers of each. It did not affect our analysis.
   1. International organization providing external financing for health
   2. Other international organization
   3. Government in receipt of external financing for health
   4. Government providing external financing for health
   5. Academic / Commentator / Consultant
   6. Civil society organization
   7. Student
   8. Other (please specify)
